# Phospholipase D1 Mediates AMP-Activated Protein Kinase Signaling for Glucose Uptake

**DOI:** 10.1371/journal.pone.0009600

**Published:** 2010-03-09

**Authors:** Jong Hyun Kim, Ji-Man Park, Kyungmoo Yea, Hyun Wook Kim, Pann-Ghill Suh, Sung Ho Ryu

**Affiliations:** 1 Division of Molecular and Life Sciences, Pohang University of Science and Technology, Pohang, Kyungbuk, Republic of Korea; 2 Laboratory of Molecular Cardiology, National Heart, Lung and Blood Institute, National Institutes of Health, Bethesda, Maryland, United States of America; University of Pittsburgh, United States of America

## Abstract

**Background:**

Glucose homeostasis is maintained by a balance between hepatic glucose production and peripheral glucose utilization. In skeletal muscle cells, glucose utilization is primarily regulated by glucose uptake. Deprivation of cellular energy induces the activation of regulatory proteins and thus glucose uptake. AMP-activated protein kinase (AMPK) is known to play a significant role in the regulation of energy balances. However, the mechanisms related to the AMPK-mediated control of glucose uptake have yet to be elucidated.

**Methodology/Principal Findings:**

Here, we found that AMPK-induced phospholipase D1 (PLD1) activation is required for ^14^C-glucose uptake in muscle cells under glucose deprivation conditions. PLD1 activity rather than PLD2 activity is significantly enhanced by glucose deprivation. AMPK-wild type (WT) stimulates PLD activity, while AMPK-dominant negative (DN) inhibits it. AMPK regulates PLD1 activity through phosphorylation of the Ser-505 and this phosphorylation is increased by the presence of AMP. Furthermore, PLD1-S505Q, a phosphorylation-deficient mutant, shows no changes in activity in response to glucose deprivation and does not show a significant increase in ^14^C-glucose uptake when compared to PLD1-WT. Taken together, these results suggest that phosphorylation of PLD1 is important for the regulation of ^14^C-glucose uptake. In addition, extracellular signal-regulated kinase (ERK) is stimulated by AMPK-induced PLD1 activation through the formation of phosphatidic acid (PA), which is a product of PLD. An ERK pharmacological inhibitor, PD98059, and the PLD inhibitor, 1-BtOH, both attenuate ^14^C-glucose uptake in muscle cells. Finally, the extracellular stresses caused by glucose deprivation or aminoimidazole carboxamide ribonucleotide (AICAR; AMPK activator) regulate ^14^C-glucose uptake and cell surface glucose transport (GLUT) 4 through ERK stimulation by AMPK-mediated PLD1 activation.

**Conclusions/Significance:**

These results suggest that AMPK-mediated PLD1 activation is required for ^14^C-glucose uptake through ERK stimulation. We propose that the AMPK-mediated PLD1 pathway may provide crucial clues to understanding the mechanisms involved in glucose uptake.

## Introduction

Circulating glucose levels reflect a balance between glucose production by the liver and glucose utilization in skeletal muscles [1], [2]. Energy deprivation occurs when cellular glucose levels are depleted by nutritional and environmental stressors such as glucose starvation, pressure overload, oxidative stress or hypoxia [3]–[8]. Once the balance breaks down, regulatory proteins such as AMPK are stimulated to restore it. AMPK, a serine/threonine protein kinase, is known to play an important role in the regulation of glucose uptake [9].

AMPK is often referred to as an energy sensor because it maintains the balance of AMP:ATP ratios and its activity increases with decreasing levels of cellular ATP. Thus, AMPK turns on ATP-producing catabolic pathways and turns off ATP-consuming processes under energy deprivation conditions [10]. AMPK is a heterotrimeric protein complex composed of one catalytic subunit (α) and two regulatory subunits (β and γ) Its activity is also regulated by both AMP and the tumor suppressor, LKB1. Regulation by both AMP and LKB1 accompanies the interaction with AMPK and its phosphorylation [2]. LKB1, a serine-threonine kinase, is the most well characterized upstream kinase for AMPK activation. LKB1 phosphorylates a conserved Thr-172 within the T-loop of the AMPK catalytic subunit (α), leading to its activation. Activated AMPK induces phosphorylation at the Ser-79 site of acetyl-CoA carboxylase (ACC) as a direct downstream target. ACC regulates the conversion of acetyl-CoA to malonyl-CoA in the *de novo* lipid synthesis pathway [2]. Recently, several groups have reported that AMPK plays a major role in the regulation of metabolic stress-induced glucose uptake in both heart and skeletal muscles [11]–[13]. Activation of AMPK by AICAR has also been shown to increase glucose uptake via a phosphoinositide-3 kinase (PI-3K)-independent mechanism [14]. However, until now, the downstream target molecules of AMPK-mediated glucose uptake have been largely unknown.

Phospholipase D (PLD) is a ubiquitous enzyme that catalyzes the hydrolysis of phosphatidylcholine (PC) to produce metabolically active phosphatidic acid (PA). PLDs are membrane-bound proteins and the relative distributions of PLD isoforms (PLD1 and PLD2) are distinct in various cell types [15]–[16]. Consistent with its diverse locations, PLD exerts multiple cellular functions in different cell types [17]–[19]. Previous studies have suggested that PLD activity is regulated by various stress signals including serum withdrawal, glucose availability, oxidative stress and pressure overload [20]–[22]. As a result, it has been proposed that PLD plays a role under stressful conditions. However, the regulatory mechanisms involved in PLD activity for glucose uptake under glucose deprivation conditions in skeletal muscle cells have not been studied to date.

In this study, we attempted to gain insight into the relationships between AMPK, PLD1 and glucose uptake. Based on the results of the present study, we suggest that AMPK-induced PLD1 activity is required for the regulation of glucose uptake via ERK activation in muscle cells and that PLD1 has a novel site that is phosphorylated by AMPK. Taken together, these findings imply that the AMPK-PLD1 pathway may contribute to the control of glucose homeostasis.

## Results

### AMPK Stimulates PLD1 Activity

**Figure 1:**
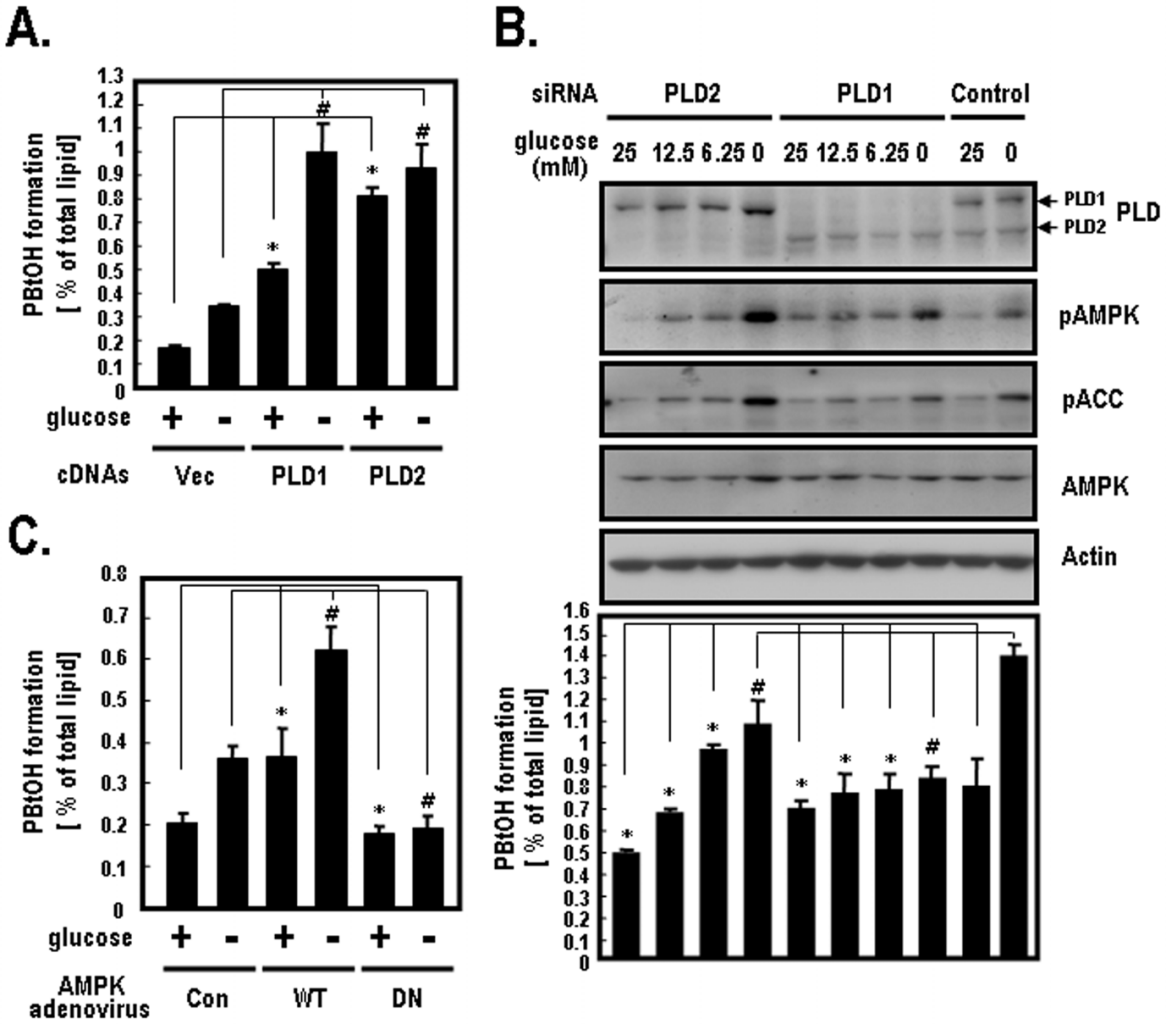
The effects of glucose deprivation on PLD activity. **A.** HEK-293 cells were transfected with the indicated plasmids for vector, PLD1 or PLD2. At 40 h after transfection, cells were incubated in glucose free-DMEM with or without 25 mM glucose for 2 h. Cells were then labeled with [^3^H]myristric acid for 4 h and the formation of [^3^H]PBtOH was quantified as described in the materials and methods. Quantitative data represent the means of three independent experiments. *, *p*<0.05 versus vector-transfected cells with glucose; #, *p*<0.05 versus vector-transfected cells without glucose. **B.** HEK-293 cells were transfected with siRNAs for PLD1, PLD2 or control (Luciferase). At 40 h after transfection, cells were incubated in glucose free-DMEM with the indicated concentrations of glucose (25 mM [4.5 g/L], 12.5 mM [2.25 g/L], 6.25 mM [1.125 g/L]) for 2 h. Cells were then labeled with [^3^H]myristric acid for 4 h and the formation of [^3^H]PBtOH was quantified. The expression levels of PLD and AMPK were analyzed by western blotting using antibodies directed against PLDs or AMPK. pACC (Ser-79) and pAMPK (Thr-172) were used as positive controls to show the activation of AMPK pathway induced by glucose deprivation. Actin was used as a loading control. Quantitative data represent the means of three independent experiments. *, *p*<0.05 versus control siRNA-transfected cells in the presence of glucose; #, *p*<0.05 versus control siRNA-transfected cells in the absence of glucose. **C.** HEK-293 cells were infected with control (empty vector), AMPK-WT or AMPK-DN adenovirus. At 40 h after infection, cells were incubated in glucose free-DMEM with or without 25 mM glucose for 2 h. Cells were labeled with [^3^H]myristric acid for 4 h and the formation of [^3^H]PBtOH was then quantified. Data represent the means of three independent experiments. *, *p*<0.05 versus control adenovirus-infected cells with glucose; #, *p*<0.05 versus control adenovirus-infected cells without glucose.

Previous studies have shown that PLD activity was regulated when energy imbalance was induced by serum withdrawal or glucose starvation [22], [23]. To determine the specific role of PLD isoforms under glucose deprivation, we examined PLD activity after the ectopic expression of PLD1 or PLD2 isoforms in HEK-293 cells. Endogenous PLD activity led to a significant increase in vector-transfected cells under glucose deprivation, as indicated by increased byproducts (PBtOH) of PLD-mediated transphosphatidylation (Figure 1A). PLD1 activity increased approximately 2-fold in PLD1-transfected cells under glucose deprivation conditions, whereas PLD2 activity changed very slightly under the same conditions (Figure 1A). Since, it is well known that AMPK is regulated by changes in the physiological glucose concentration [4], [9], we investigated the relationship between PLD isoforms and AMPK under different glucose concentrations. We first silenced PLD1 or PLD2 in HEK-293 cells and then examined PLD activity. As shown in Figure 1B, endogenous PLD1 activity was enhanced in PLD2-silenced cells in response to decreasing glucose concentrations, whereas endogenous PLD2 activity was unaffected in PLD1-silenced cells. The expression levels of PLD, AMPK, pACC (Ser-79) and pAMPK (Thr-172) were analyzed by western blotting. pACC (Ser-79) and pAMPK (Thr-172) were used as positive controls to show the activation of AMPK pathway induced by glucose deprivation. Figure 1A shows that PLD2 had a higher basal activity (1.6-fold) than PLD1 in the presence of glucose (25 mM) when PLD isoforms were overexpressed in HEK-293 cells. As shown in Figure 1B, endogenous PLD2 activity in PLD1 siRNA-transfected cells was also higher than endogenous PLD1 activity in PLD2 siRNA-transfected cells in the presence of both 25 mM and 12.5 mM glucose. Substantially, the basal activities of both PLD1 and PLD2 are quite different since there are distinct regulatory mechanisms of PLD isoforms. Additionally, it has previously been shown that the conditions used in Figure 1A and B do not lead to saturation of PLD2 isoform. Therefore, these results indicate that the activity of PLD1, but not that of PLD2, is inversely dependent upon the glucose concentration. Furthermore, to determine if the stimulation of PLD is regulated by AMPK activity, we infected HEK-293 cells with AMPK adenoviruses using AMPK-wild type (WT) and AMPK-dominant negative (DN). In control adenovirus-infected cells, PLD activity significantly increased when glucose was deprived. In AMPK-WT adenovirus-infected cells, PLD activity increased approximately 1.8-fold in the presence of glucose when compared to control adenovirus-infected cells. Under glucose deprivation, PLD activity increased about 1.7-fold in AMPK-WT, whereas no change in the PLD activity in AMPK-DN was observed (Figure 1C). These results suggest that PLD activity is regulated by AMPK activation.

### AMPK Directly Phosphorylates PLD1

**Figure 2:**
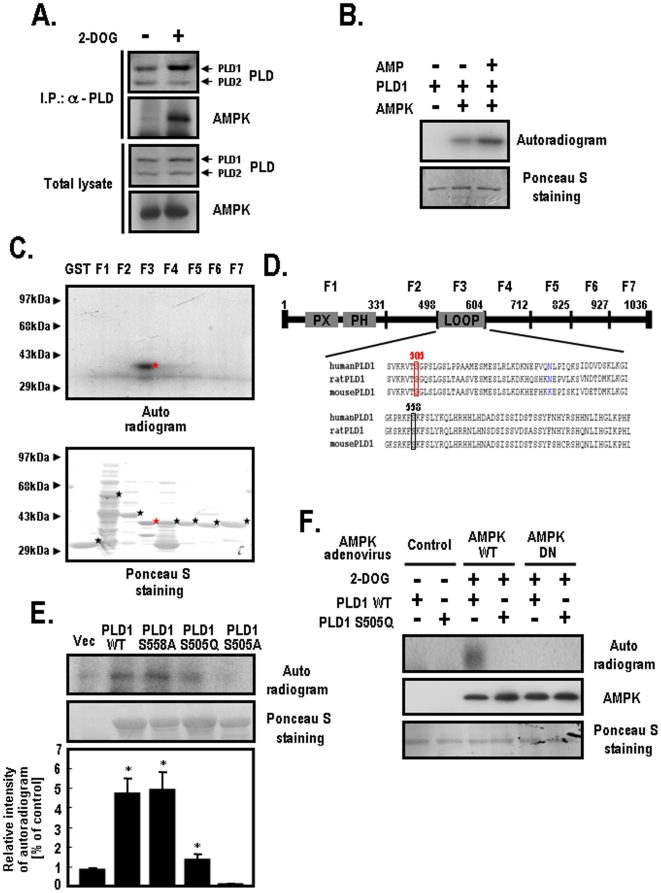
AMPK phosphorylates PLD1 but not PLD2. **A.** After treatment with 25 mM 2-DOG for 10 min, HEK-293 cells were lysed with buffer containing 1% Triton X-100 and 1% cholic acid. After determination of the total protein concentrations, equal amounts of extracts were immunoprecipitated with anti-PLD antibody. After brief centrifugation and washing, the precipitates were subjected to SDS-PAGE and immunoblotted with anti-PLD and anti-AMPK antibodies. The total lysates show approximately 1% of the proteins used for immunoprecipitation. **B.** AMPK was incubated with purified PLD1 (100 ng) in kinase buffer A (50 mM Tris/HCl, pH 7.4, 1 mM dithiothreitol, 0.02% Brij-35 and 10 µCi of [γ-^32^P]ATP (3,000 Ci/mmol)) in the presence or absence of AMP (200 nM). This reaction was terminated by the addition of SDS sample buffer. The precipitates were then subjected to SDS-PAGE and exposed to photographic film for autoradiography. Ponceau S staining shows the protein amounts in each sample. **C.** GST-truncated PLD1 fusion mutants were expressed in bacteria and then purified using glutathione sepharose beads. The truncated PLD1 mutants were followed by F1 (amino acid [a.a.] 1 to 331), F2 (a.a. 332 to 498), F3 (a.a. 499 to 604), F4 (a.a. 605 to 712), F5 (a.a. 713 to 825), F6 (a.a. 826 to 927) and F7 (a.a. 928 to 1036). AMPK was incubated with an equal amount of each GST-truncated PLD1 fusion mutants (1 µg) in kinase buffer A in the presence of AMP (200 nM). This reaction was terminated by the addition of SDS sample buffer and exposed to photographic film for autoradiography. **D.** Schematic diagram of the PLD1 sequences. Human PLD1 sequences corresponding to the loop region were aligned with those of rat and mouse. Candidate phosphorylation sites of PLD1 for AMPK are shown in the boxes. **E.** HEK-293 cells were transfected with the indicated plasmids for vector, PLD1-WT, S558A, S505Q or S505A. At 48 h after transfection, cells were lysed with buffer and the lysates were immunoprecipitated with anti-PLD antibody. After brief centrifugation and washing, AMPK was incubated with the PLD1-immunocomplexes in kinase buffer A. This reaction was terminated by the addition of SDS sample buffer and the precipitates were then subjected to SDS-PAGE and exposed to photographic film for autoradiography. The relative intensity of the autoradiogram was quantified as described in the materials and methods. The data shown represent one of three independent experiments. Bar graphs reflect the average of three independent experiments. *, *p*<0.05 versus vector-transfected cells. **F.** HEK-293 cells were transfected with the indicated plasmids of PLD1-WT or PLD1-S505Q. At 24 h post-transfection, cells were infected with control (empty vector), AMPK-WT or AMPK-DN adenovirus. At 24 h after infection, cells were loaded with [^32^P] orthophosphate (3 mCi/ml) for 5 h and then treated with 25 mM of 2-DOG for 10 min. Next, the extracts were prepared with lysis buffer and immunoprecipitated with anti-PLD antibody. After brief centrifugation and washing, the precipitates were then subjected to SDS-PAGE, transferred to nitrocellulose membranes and exposed to photographic film for autoradiography. The expression levels of AMPK and PLD1 were analyzed by western blotting and ponceau S staining. Data represent one of three independent experiments.

To delineate the regulatory mechanism of PLD1 by AMPK, we performed co-immunoprecipitation experiments using HEK-293 cells, which express PLD1 as a major isoform. When 2-deoxyglucose (2-DOG) was used as a glucose mimic to induce ATP depletion, endogenous PLD was physically associated with AMPK in a 2-DOG-dependent manner (Figure 2A). To determine if AMPK directly phosphorylates PLD1, we incubated PLD1 and AMPK with or without AMP. As shown in Figure 2B, AMPK phosphorylated the purified PLD1 *in vitro*. Specifically, AMPK enhanced the phosphorylation of PLD1 in the presence of AMP. However, AMPK was unable to phosphorylate the purified PLD2 directly in the presence of AMP (data not shown). To verify the potential phosphorylation sites, we performed *in vitro* phosphorylation assays using GST-truncated PLD1 fusion mutants. Figure 2C demonstrates that the F3 fragment corresponding to amino acid region 499–604 was specifically phosphorylated by AMPK in the presence of AMP. Based on this finding, we surveyed the candidate phosphorylation sites of the PLD1-loop corresponding to the 499–604 amino acid region by comparing it with an AMPK consensus motif. Interestingly, the PLD1-loop contained two candidate phosphorylation sites including the Ser-505 and the Ser-558 residues (Figure 2D). However, no possible phosphorylation sites were observed in the corresponding region of the PLD2 sequence because the central loop is a unique region of PLD1. To define the phosphorylation sites of PLD1, we generated several constructs including PLD1-S505A, PLD1-S505Q and PLD1-S558A. As shown in quantified results of Figure 2E, both PLD1-WT and PLD1-S558A were phosphorylated by AMPK in the presence of AMP whereas both PLD1-S505A and PLD1-505Q were not phosphorylated under the same conditions. We also utilized AMPK adenovirus to determine if AMPK phosphorylates PLD1 on the Ser-505 residue *in vivo*. After treatment with 2-DOG, PLD1 was phosphorylated by AMPK-WT, whereas PLD1-S505Q remained unphosphorylated (Lane 3 and 4 in Figure 2F). These findings demonstrate that AMPK phosphorylates PLD1 on the Ser-505 residue *in vivo*. Based on these results, we suggest that PLD1 may be a novel substrate of AMPK.

### AMPK Regulates PLD1 Activity through Phosphorylation

**Figure 3:**
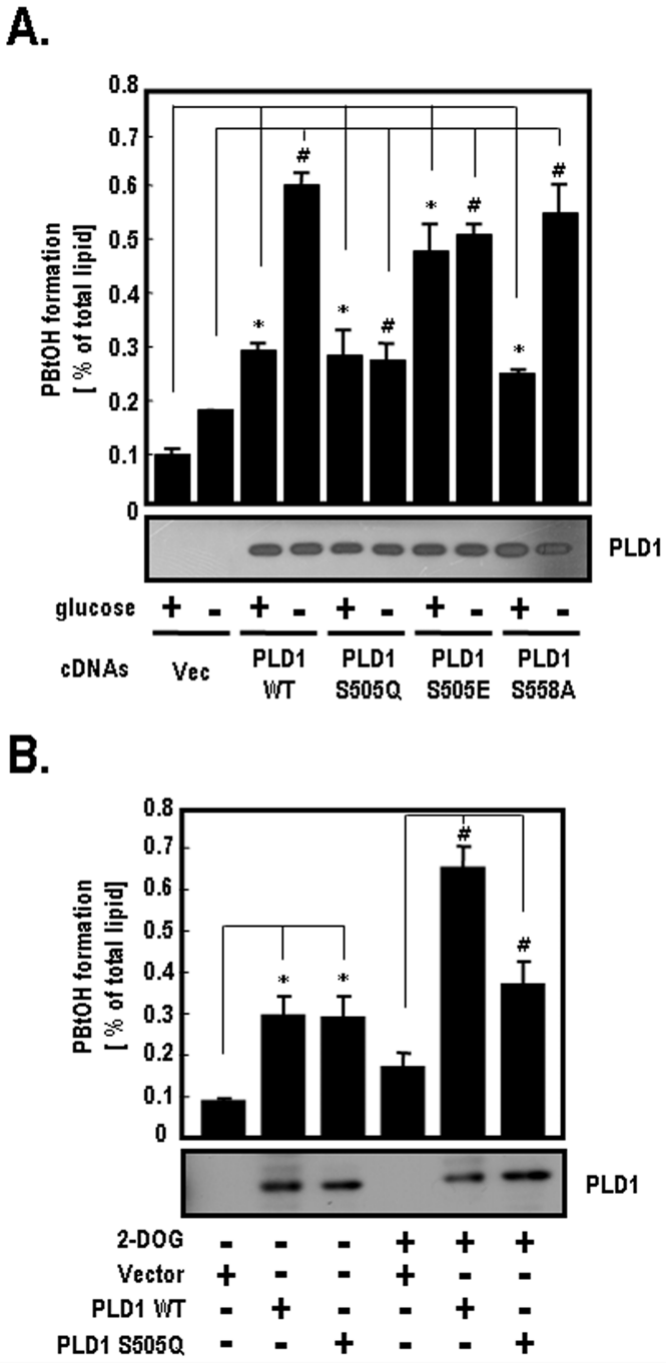
Activity of PLD1-WT and PLD1-mutants under glucose deprivation. **A.** HEK-293 cells were transfected with the indicated plasmids of vector, PLD1-WT, S558A, S505Q or S505E. At 40 h after transfection, cells were incubated in glucose free-DMEM with or without 25 mM glucose for 2 h. PLD activity in each group was then measured by the formation of [^3^H]PBtOH. The expression levels of PLD1 were analyzed by western blotting. Quantitative data represent the means of four independent experiments. *, *p*<0.01 versus vector-transfected cells with glucose; #, *p*<0.01 versus vector-transfected cells without glucose. **B.** HEK-293 cells were transfected with siRNAs directed against PLD. At 24 h post-transfection, cells were transfected with siRNA-resistant forms of the indicated PLD1 [45]. After 24 h, cells were treated with 25 mM of 2-DOG for 10 min and the formation of [^3^H]PBtOH was then quantified as previously described. Re-expression levels of PLD1 were analyzed by western blotting. Quantitative data represent the means of three independent experiments. *, *p*<0.01 versus vector-transfected cells without 2-DOG; #, *p*<0.01 versus vector-transfected cells with 2-DOG.

To determine if PLD1 activity is substantially regulated by phosphorylation, we measured the PLD1 activity using several PLD1-mutants including PLD1-WT, S505Q, S505E and S558A in the presence or absence of 25 mM glucose. As shown in Figure 3A, PLD1-WT activity was stimulated by the removal of glucose, whereas PLD1-S505Q activity was not. PLD1-S505Q still showed a basal level of activity similar to that of PLD1-WT in the presence of glucose. However, PLD1-S505A had no basal activity, regardless of the glucose concentrations (data not shown). These results indicate that the substitution of Ser to Ala on the Ser-505 residue is related to the basal activation of PLD1. In addition, PLD1-S505E displayed much higher activity than PLD1-S505Q, regardless of the glucose concentrations. PLD1-S558A activity increased by approximately 2.2-fold under glucose deprivation conditions. PLD1-WT activity enhanced about 2.1-fold under the same conditions. Based on these findings, the Ser-558 residue of PLD1 does not appear to be a regulatory site that is phosphorylated by AMPK. To confirm that PLD1 activity is regulated by ATP depletion, we measured the PLD1 activity after treatment with 2-DOG. As shown in Figure 3B, the PLD1-WT activity was stimulated by ATP depletion induced by 2-DOG, but PLD1-S505Q was unaffected under the same conditions. Taken together, these results suggest that AMPK stimulates PLD1 activity through phosphorylation of the Ser-505 residue under glucose deprivation.

### AMPK-Induced PLD1 Activation Is Required for ERK Stimulation

**Figure 4:**
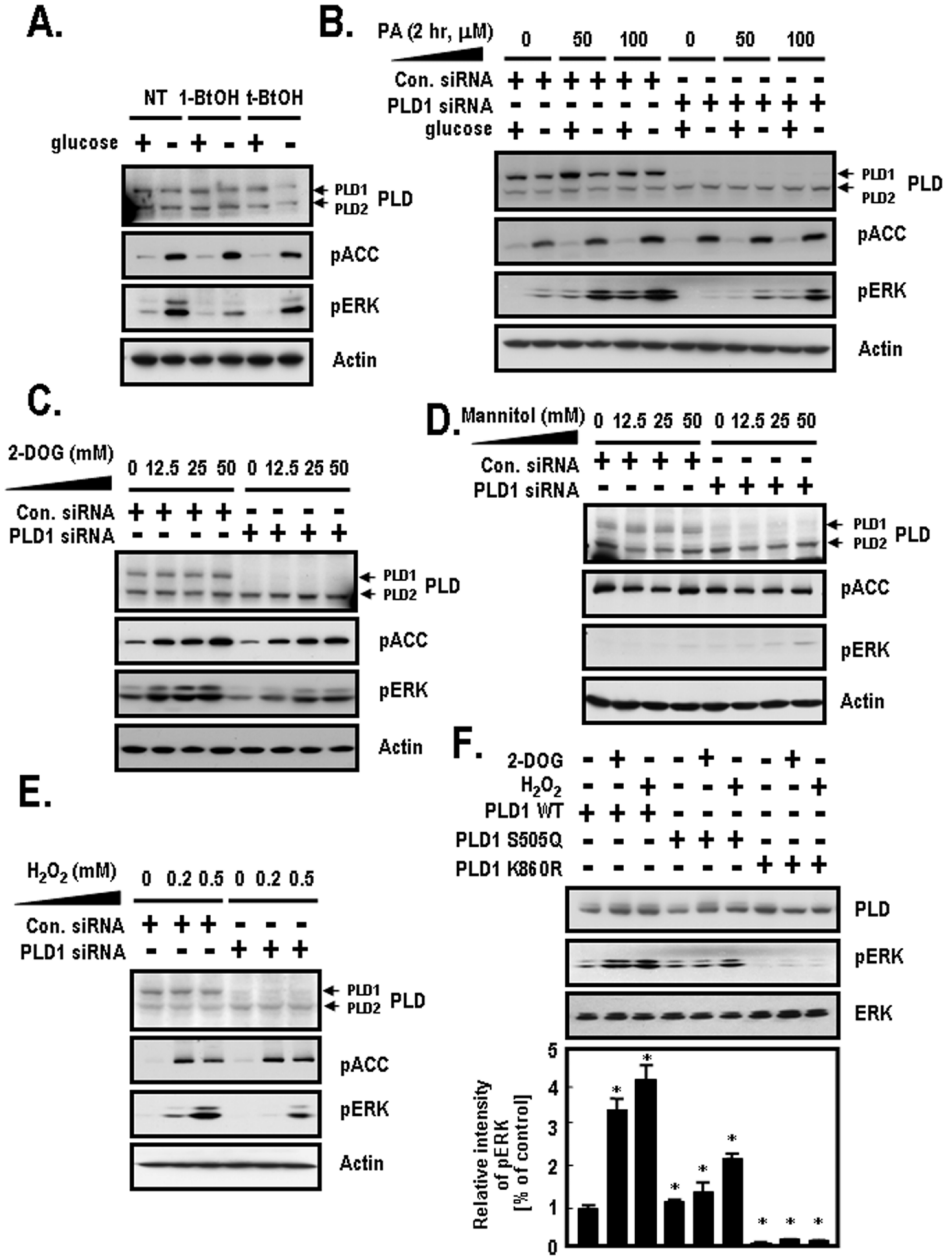
The effects of PLD1 on ERK activation by various stimuli. **A.** After HEK-293 cells were incubated in glucose free-DMEM with or without 25 mM glucose for 2 h, cells were treated with 1-BtOH or t-BtOH for 15 min and then lysed with lysis buffer. Cell lysates were then analyzed by western blotting with the indicated antibodies. pACC (Ser-79) was used as a positive control to show the activation of AMPK pathway induced by glucose deprivation. Actin was used as a loading control. These data represent the mean of three independent analyses. **B.** HEK-293 cells were transfected with the indicated siRNAs of control (Luciferase) or PLD1. At 48 h after transfection, cells were incubated in glucose free-DMEM with or without 25 mM glucose and the indicated concentrations of exogenous PA for 2 h. Cells were harvested and then lysed with lysis buffer. pACC (Ser-79) was used as a positive control and the protein amounts were normalized against actin. Cell lysates were subjected to SDS-PAGE and analyzed by western blotting with the indicated antibodies. These data represent one of three experiments in independent analyses. **C.** HEK-293 cells were transfected with the indicated siRNAs of control (Luciferase) or PLD1. At 48 h after transfection, cells were treated with the indicated concentrations of 2-DOG for 10 min. Then, cells were lysed with lysis buffer and lysates were subjected to SDS-PAGE and then immunoblotted with the indicated antibodies. pACC (Ser-79) was used as a positive control to show the activation of the AMPK pathway induced by treatment with 2-DOG. Actin was used as a loading control for western blotting. The data shown represent one of three independent experiments. **D.** HEK-293 cells were transfected with the indicated siRNAs for control (Luciferase) or PLD1. At 48 h after transfection, cells were treated with the indicated concentrations of mannitol as a negative control to induce hyperosmolar stress for 10 min. Cells were prepared according to the methods described in panel **A** and cell lysates were then immunoblotted with the indicated antibodies. These data shown are from one of three independent experiments. **E.** HEK-293 cells were transfected with the indicated siRNAs of control (Luciferase) or PLD1. At 48 h after transfection, cells were treated with the indicated concentrations of H_2_O_2_ for 10 min as a positive control to induce oxidative stress. Cells were prepared according to the methods described in panel **A** and cells lysates were then immunoblotted with the indicated antibodies. pACC (Ser-79) was used as a positive control to show the activation of the AMPK pathway induced by H_2_O_2_ treatment. Actin was used as a loading control for western blotting. These data shown are from one of three independent experiments. **F.** HEK-293 cells were transfected with the indicated plasmids for PLD1-WT, PLD1-S505Q or PLD1-K860R. At 48 h after transfection, cells were treated with 25 mM of 2-DOG or 0.2 mM of H_2_O_2_ for 10 min. Cell lysates were prepared as described in **A** and then immunoblotted with the indicated antibodies. pERK activation was normalized against the expression of ERK. The relative intensity of pERK was quantified as described in the materials and methods. The data shown represent one of three independent experiments. Bar graphs reflect the average of three independent experiments. *, *p*<0.05 versus PLD1-WT-transfected cells.

Based on the finding that AMPK stimulates PLD1 through direct phosphorylation (Figures 1 and 2), we investigated the downstream target molecules of AMPK-induced PLD1 activation to determine the reasons for PA formation. Previous studies have found that the activation of PLD may implicate the mitogen activated protein kinase (MAPK) pathway through raf-1 activation via recruitment of PLD-generated PA binding [24], [25]. We attempted to determine if stimulation of ERK is regulated by PLD activity under glucose deprivation conditions. To accomplish this, we utilized 1-butanol (1-BtOH) as an inhibitor of PLD and t-butanol (t-BtOH) as a negative control in the presence or absence of glucose. 1-butanol was used to prevent generation of the PLD products, PA. ERK activation, which was determined based on an increase in the phosphorylated ERK levels, was dramatically increased by glucose deprivation in both NT (not treated)-cells and t-BtOH-treated cells (Figure 4A). However, ERK activation was very slightly increased in 1-BtOH-treated cells under the same conditions. In the absence of glucose, ERK activation was attenuated in 1-BtOH-treated cells when compared to both NT-cells and t-BtOH-treated cells. pACC (Ser-79) was used as a positive control to show the activation of the AMPK pathway induced by glucose deprivation. These results indicate that PLD activation is required for ERK activation. To elucidate the effects of PLD1-generated PA on ERK activation, we silenced PLD1 with siRNA and then treated the cells with various concentrations of exogenous PA. As shown in Figure 4B, ERK activation was attenuated in PLD1 siRNA-transfected cells when compared to control siRNA-transfected cells. In addition, exogenous PA increased ERK activation in a dose dependent manner. These results imply that PA supplied by PLD activation is responsible for ERK activation. Next, we examined the specificity of ERK activation by the presence of various extracellular stressors including 2-DOG, mannitol and H_2_O_2_. ERK activation was reduced in PLD1 siRNA-transfected cells in response to 2-DOG when compared to control siRNA-transfected cells (Figure 4C). To exclude the influence of nonspecific 2-DOG by hyperosmolar stress, we utilized mannitol as a negative control to induce hyperosmolar stress. As shown in Figure 4D, ERK activation was slightly increased in PLD1 siRNA-transfected cells in response to treatment with mannitol when compared to control siRNA-transfected cells. These results imply that PLD1 activation induced by energy depletion but not hyperosmolar stress is required for ERK activation. H_2_O_2_ used as a positive control to induce oxidative stress also resulted in a reduction of ERK activation in PLD1 siRNA-transfected cells when compared to control siRNA-transfected cells (Figures 4E). To confirm the effects of PLD lipase activity on ERK activation, we included another construct, PLD1-K860R, which is a lipase-inactive mutant. As shown in Figure 4F, PLD1-WT showed approximately 3.3-fold greater levels of ERK activation in response to 2-DOG and about 4.2-fold greater ERK activation following H_2_O_2_ treatment. The ERK activation observed in PLD1-WT was similar to the activation observed in PLD1-S505Q under basal conditions (Lanes 1 and 4). PLD1-S505Q also showed approximately 1.1-fold greater levels of ERK activation in response to treatment with 2-DOG and about 2-fold greater ERK activation following H_2_O_2_ treatment. PLD1-K860R showed decreased ERK activation under basal conditions when compared to PLD1-WT (Lanes 1 and 7). PLD1-K860R showed very slightly higher ERK activation, despite treatment with 2-DOG or H_2_O_2_. Based on these observations, we suggest that ERK activation is dependent on PLD1 activity in response to 2-DOG or H_2_O_2_. Therefore, these results imply that AMPK-mediated PLD1 activation contributes to the stimulation of ERK under energy depletion conditions caused by glucose deprivation and oxidative stress.

### AMPK-Induced PLD1 Activation Is Important for ^14^C-Glucose Uptake in Differentiated C2C12 and L6 GLUT4-myc Myotube Cells

**Figure 5:**
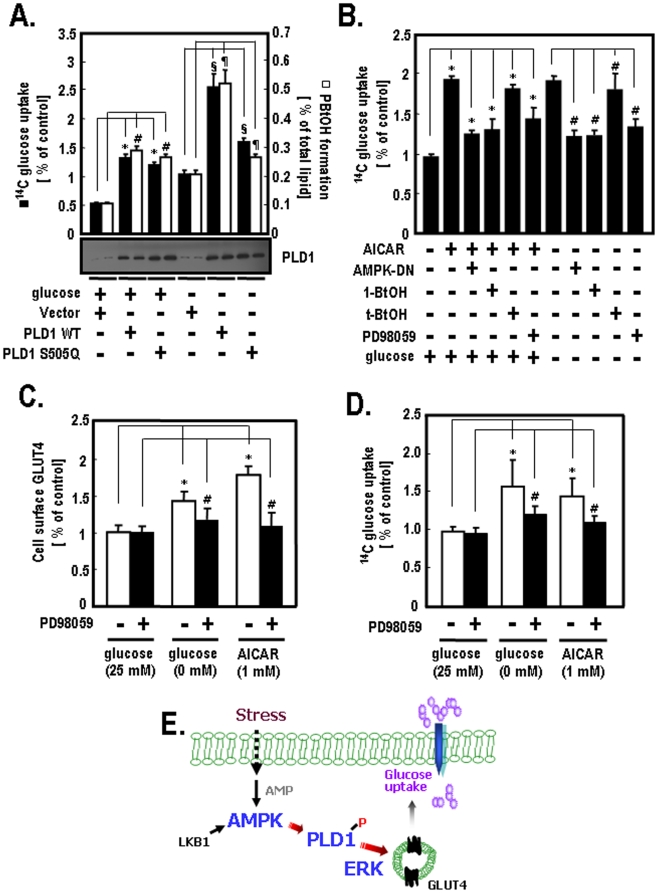
Glucose uptake in differentiated C2C12 and L6 GLUT4-myc myotube cells. **A.** C2C12 cells were transfected with PLD siRNAs. At 24 after transfection, cells were transfected with the indicated siRNA-resistant PLD1 plasmids for vector, PLD1-WT or PLD1-S505Q [45]. After 24 h, cells were incubated in glucose free-DMEM with or without 25 mM glucose for 2 h. The expression levels of PLD1 were then analyzed by western blotting. The amounts of ^14^C-glucose uptake and [^3^H]PBtOH formation were analyzed as described in the materials and methods. The quantitative data shown represent the means of three independent experiments. *, #, *p*<0.05 versus vector-transfected cells with glucose; ¶, §, *p*<0.05 versus vector-transfected cells without glucose. **B.** After C2C12 cells were differentiated according to the procedures described in the materials and methods, cells were incubated in glucose free-DMEM with or without 25 mM glucose for 2 h. Cells were then treated with the indicated exogenous agents (AICAR [1 mM], 1-BtOH [0.4%], t-BtOH [0.4%], PD98059 [20 µM]) for 30 min. For the case of infection of AMPK-DN adenovirus, the differentiated C2C12 cells were incubated with the virus for 48 h. Cells were then incubated in glucose free-DMEM with or without 25 mM glucose for 2 h and were treated with 1 mM of AICAR for 15 min. The uptake of ^14^C-glucose was measured and quantified. The quantitative data shown represent the means of four independent experiments. *, *p*<0.05 versus vehicle treatment in the presence of glucose; #, *p*<0.05 versus vehicle treatment in the absence of glucose. **C.** L6 GLUT4-myc myoblast cells were differentiated according to the procedures described in the materials and methods. The differentiated L6 GLUT4-myc myotube cells were incubated in glucose free-DMEM with or without 25 mM glucose for 2 h or pre-treated with 1 mM AICAR for 30 min. After treatment with 20 µM of PD98059 for 30 min, the amount of cell surface GLUT4 was quantified as described in the materials and methods. This experiment was performed as three independent assays. *, *p*<0.01 versus vehicle treatment in the presence of 25 mM glucose; #, *p*<0.01 versus PD98059 treatment in the presence of 25 mM glucose. **D.** The differentiated L6 GLUT4-myc myotube cells were incubated in glucose free-DMEM with or without 25 mM glucose for 2 h or pre-treated with 1 mM AICAR for 30 min. After treatment with 20 µM of PD98059 for 30 min, the uptake of ^14^C-glucose was measured and quantified. Quantitative data represent the means of three independent experiments. *, *p*<0.01 versus vehicle treatment in the presence of 25 mM glucose; #, *p*<0.01 versus PD98059 treatment in the presence of 25 mM glucose. **E.** A hypothetical model. This diagram shows a hypothetical model of extracellular stress-mediated glucose uptake. Glucose uptake is induced by the AMPK-activated signaling pathway. In this pathway, AMPK increases PLD1 activity through phosphorylation and promotes glucose uptake by triggering subsequent ERK activation in muscle cells.

Previous studies have reported that AMPK activity is involved in glucose homeostasis in a variety of cells [1], [10], and that PLD activity is required for glucose uptake in muscles and adipocytes [21], [23], [26]. Based on these previous findings, we investigated the mechanisms by which AMPK and PLD regulate the glucose uptake using 2-deoxy[^14^C-glucose] as an indicator for the glucose uptake assay. As shown in Figure 5A, PLD1-WT showed an approximately 2.8-fold increase in ^14^C-glucose uptake in the presence of glucose when compared to vector-transfected cells (Columns 1 and 2). PLD1-S505Q showed about 2.7-fold greater in ^14^C-glucose uptake under the same conditions (Columns 1 and 3). PLD1-WT showed an approximately 1.9-fold increase in ^14^C-glucose uptake under glucose deprivation conditions (Columns 2 and 5), while PLD1-S505Q showed only a 1.2-fold increase in ^14^C-glucose uptake under the same conditions (Columns 3 and 6). The changes in ^14^C-glucose uptake in response to glucose deprivation showed a similar pattern to that of PLD1 activity. These results indicate that the Ser-505 residue of PLD1 is required for ^14^C-glucose uptake in response to glucose deprivation. Next, we measured the ^14^C-glucose uptake in response to AICAR (used as an AMPK activator) and AMPK-DN to determine the effects of AMPK on glucose uptake. As shown in Figure 5B, the ^14^C-glucose uptake was increased by about 1.9-fold by AICAR, but was significantly reduced by AMPK-DN (Columns 2 and 3). To examine the effects of PLD and ERK on glucose uptake, we treated the cells with 1-BtOH (a PLD inhibitor) and PD98059 (an ERK inhibitor). The ^14^C-glucose uptake was attenuated in response to treatment with 1-BtOH as well as PD98059. Furthermore, the increase in ^14^C-glucose uptake regulated by AICAR was correlated with that of the ^14^C-glucose uptake induced by glucose deprivation (Columns 2 and 7). The increase in ^14^C-glucose uptake induced by glucose deprivation was decreased by AMPK-DN, 1-BtOH and PD98059 (Columns 8, 9 and 11). Taken together, these results imply that AMPK-PLD1-induced ERK activation is necessary for glucose uptake in differentiated C2C12 cells. To further define the role of ERK activation in glucose uptake, we investigated the amount of cell surface glucose transport (GLUT) 4 and the ^14^C-glucose uptake in differentiated L6 GLUT4-myc myotube cells. The levels of cell surface GLUT4 were enhanced by about 1.5-fold by glucose deprivation when compared to the presence of glucose. AICAR increased the amount of cell surface GLUT4 by approximately 1.8-fold. However, the levels of cell surface GLUT4 induced by both glucose deprivation and AICAR were attenuated by the presence of PD98059 (Figure 5C). As shown in Figure 5D, glucose deprivation increased the ^14^C-glucose uptake by approximately 1.6-fold in the absence of PD98059 when compared to the presence of 25 mM glucose (Columns 1 and 2). AICAR enhanced the ^14^C-glucose uptake by about 1.5-fold under the same conditions (Columns 1 and 3). However, the ^14^C-glucose uptake stimulated by both glucose deprivation and AICAR were attenuated by the presence of PD98059. Taken together, these results suggest that the stimulation of ERK is required for glucose uptake in differentiated L6 GLUT4-myc myotube cells.

## Discussion

Although it is known that extracellular stressors such as AICAR increase glucose uptake through the AMPK pathway in muscle cells, the downstream aspects of AMPK signaling are largely unknown. In the present study, we found that PLD1 is a novel signaling effector of AMPK. Several lines of evidence support the notion that PLD1-mediated AMPK activation is required for ERK activation and glucose uptake in muscle cells (Figure 5E). For example, AMPK stimulates PLD1 through phosphorylation as demonstrated by a reduction of PLD1 activity in AMPK-DN adenovirus-infected cells (Figure 1). Moreover, mutagenesis and *in vitro* and *in vivo* phosphorylation assays demonstrated that AMPK phosphorylates PLD1 on the Ser-505 (Figure 2). ERK activation was found to be dependent on energy status, but not hyperosmolar stresses (Figure 4). Finally, ERK activation by AMPK-mediated PLD1 was found to be essential for ^14^C-glucose uptake in differentiated C2C12 and L6 GLUT4-myc myotube cells (Figure 5). Taken together, these results suggest that PLD1 is a downstream effector of AMPK in a signaling pathway related to glucose uptake under glucose deprivation conditions in muscle cells, C2C12 and L6 GLUT4-myc myotube cells. Understanding the molecular mechanism involved in glucose uptake may provide insight into the regulation of glucose homeostasis and shed light on diseases related to type 2 diabetes.

Emoto and Huang previously reported that the expression of PLD1 facilitates insulin-stimulated glucose uptake in 3T3L1 adipocytes [26], [27]. However, their studies were limited in that they did not identify the upstream regulator of PLD1 and its roles were only studied in adipocytes. In general, muscle is known to respond to acute and transient glucose deprivation, while adipose tissue responds to chronic reactions. In the present study, we demonstrated that PLD1 phosphorylation is responsible for acute signaling mechanisms in muscle cells and that AMPK is a novel regulator of PLD1 that functions in a glucose dependent manner.

Despite extensive research on upstream stimuli that activate AMPK, few studies have been conducted to investigate the downstream substrate(s) of AMPK-mediated glucose uptake. Acetyl-coA carboxylase (ACC) is a well-known AMPK substrate that is involved in lipid metabolism. Recent reports have revealed that AS 160, which is a known substrate for AKT, is also a downstream molecule of AMPK in contraction-stimulated glucose uptake [28]-[30]. However, the recent identification of multiple functions of AMPK in energy sensing pathways indicates that it plays a more comprehensive role in such pathways. In this study, energy-deficient conditions mimicked by glucose deprivation induced AMPK activation, which subsequently stimulated PLD1 lipase activity. This AMPK-mediated PLD1 activity was involved in glucose uptake under energy-depleted conditions (Figure 5). Taken together, these findings indicate that AMPK activity driven by contraction can regulate AS 160 and that AMPK stimulation induced by glucose deprivation may control PLD1. However, further investigations are needed to determine how two different effectors, AS 160 and PLD1, are distinguished by AMPK in response to specific stimuli.

AMPK plays a pivotal role in the maintenance of glucose homeostasis, showing strong responses to glucose deprivation in muscle cells. AMPK is known to have a well-conserved consensus motif for phosphorylation. It has been reported that PLD activity is regulated through phosphorylation or protein-protein interaction [2], [9]. In this study, we demonstrated for the first time that the phosphorylation of PLD1 by AMPK is important for its activity (Figure 3). The PLD1-loop region corresponding to amino acids 499–604 is directly phosphorylated by AMPK (Figure 2C) and the phosphorylation sites for AMPK are only observed in the PLD1 sequence because the loop region is a unique characteristic of PLD1. We investigated the phosphorylation sites of the PLD1-loop region using the AMPK consensus motif, [IMLVP or hydrophobic]-X-[HRK]-X-X-[ST]-X-X-X-[IMLVP or hydrophobic], through a site scan and a BLAST search [31]–[34]. Throughout the immunoprecipitation assays and *in vitro* and *in vivo* phosphorylation assays, we found that the PLD1-loop region had two phosphorylation sites and one of them (the Ser-505 residue) is actually phosphorylated by AMPK (Figures 2E and F). Although many investigators have suggested that the PLD isoforms have different regulatory mechanisms due to their different structures, the specific role of the PLD1-loop region has not yet been defined. Here, we identified the phosphorylation site within the PLD1-loop region that regulates PLD1 activity and is involved in AMPK-mediated glucose uptake. However, additional studies may be necessary to characterize the distinct roles of the PLD1-loop region in various cell types.

Insulin is known to stimulate glucose uptake in the skeletal muscle cells through PI-3K-dependent AKT activation. However, glucose uptake following extracellular stresses such as glucose deprivation and AICAR has been reported to occur independently of AKT [3], [29], [35]. Despite these reports, the detailed signaling pathway through which glucose uptake occurs in response to extracellular stress has not yet been clarified. Farese et al. showed that the activation of AMPK by AICAR induces stimulation of ERK, which belongs to the MAPK family, through a specific pathway [21], [23]. In the present study, western blotting and gene silencing with PLD1-siRNA revealed that AMPK-mediated PLD1 activity results in the generation of PA and that PLD1 stimulates ERK activity in response to glucose deprivation (Figure 4). PA has been reported to stimulate the MAPK pathway through the recruitment of raf-1 to the membrane. In addition, AMPK stimulated by AICAR, oxidative stress (H_2_O_2_) or glucose deprivation has been found to be associated with activation of the MAPK pathway [36]–[38]. These data appear to explain the difference between the two distinct coupled signaling pathways, insulin-AKT activation and glucose deprivation-ERK stimulation. However, further studies are necessary to characterize the specificity of the signaling pathways.

In this study, we demonstrated that ERK stimulated by AMPK-induced PLD1 activation regulates the translocation of GLUT4 in L6 GLUT4-myc myotube cells. Several groups have reported that ERK activation is required for increased glucose uptake through the cell surface translocation of GLUT4 by AICAR [23], [39]. PLD1 isoform is involved in the translocation of GLUT4 and PLD1-induced PA generation is also required for the trafficking mechanisms of GLUT4 during membrane fusion and docking [26]. Based on these previous reports and the results of the present study, we suggest that the AMPK-mediated PLD1 pathway increases glucose uptake through regulation of the translocation machinery of GLUT4. However, the mechanism by which ERK regulates the translocation machinery of GLUT4 is still not clear.

In conclusion, we suggest that AMPK-mediated PLD1 activity is essential for glucose uptake via ERK activation in differentiated C2C12 and L6 GLUT4-myc myotube cells. PLD1 and its phosphorylation may serve as important points in the regulation of energy exhaust caused by both glucose deprivation and extracellular stresses. Accordingly, we suggest that the physiological interaction between AMPK and PLD1 may provide clues to solving problems in metabolic syndromes such as diabetes. The AMPK-mediated PLD1 pathway may provide attractive target molecules for prospective pharmacological studies or genetic manipulation. However, further studies are necessary to understand the relationship between this signaling cascade and related diseases.

## Materials and Methods

### Materials

An enhanced chemiluminescence kit (ECL system) and glutathione Sepharose 4B were purchased from Amersham Pharmacia Biotech. Protease inhibitor cocktail, 2-deoxyglucose (2-DOG), mannitol and o-phenylenediamine dihydrochloride were obtained from Sigma. Polyclonal anti-pAMPK antibody (Thr-172), anti-pERK (Thr-202/Tyr-204) antibody and anti-ERK antibody were purchased from Cell Signaling. AMPK, anti-AMPK antibody and polyclonal anti-pACC antibody (Ser-79) were obtained from Upstate Biotechnology, Inc. Anti-actin antibody was obtained from ICN Pharmaceuticals. β-octylglucopyranoside and PD98059 were purchased from Calbiochem. Horseradish peroxidase-conjugated goat anti-rabbit IgG and goat anti-mouse IgA, IgM and IgG were obtained from Kirkegaard & Perry Laboratories. [γ-^32^P]ATP, [^3^H]myristic acid and [^32^P]orthophosphate were purchased from Perkin-Elmer Life Sciences. C6-phosphatidic acid (PA) was obtained from Avanti Lipids Inc. 2-deoxy[^14^C-glucose] was acquired from ARC. Immobilized protein A was purchased from Pierce. Glucose free-Dulbecco's modified Eagle's medium (DMEM), phosphate free-DMEM and α-MEM were obtained from Life Technologies, Inc. Polyclonal antibody for the recognition of PLD1 and PLD2 was generated as previously described [40].

### Purification of Recombinant PLD from sf9 Cells

Hexa-histidine (His_6_)-tagged PLD was expressed in *sf9* cells and then purified by Chelating Sepharose-affinity column chromatography, which was performed as previously described [40].

### Cell Culture, Differentiation and Transfection

HEK-293 cells and C2C12 cells were obtained from American Type Culture Collection (ATCC) and were maintained according to the standard ATCC protocol. HEK-293 cells and C2C12 cells were supplemented with 10% (v/v) fetal bovine serum (FBS) at 37°C in a humidified, CO_2_-controlled (5%) incubator. L6 GLUT4-myc myoblast cells were grown in α-MEM containing 10% fetal bovine serum, 50 U of penicillin/ml and 50 µg of streptomycin/ml and then maintained in a 5% CO_2_ humidified atmosphere at 37°C. To induce myogenic differentiation, C2C12 cells were grown to 70–90% confluency and then shifted from growth medium (DMEM + 10% FBS) to differentiation medium (DMEM + 2% horse serum). To induce differentiation of L6 GLUT4-myc myoblast cells, cells were diluted to a concentration of 4×10^4^/ml with α-MEM medium containing 2% FBS and then seeded in a 24 well plate. The plate was then incubated in a 5% CO_2_ humidified atmosphere at 37°C and the medium was changed every other day. After 7 days, L6 GLUT4-myc myoblast cells had differentiated into myotubes. HEK-293 cells were transfected using Lipofectamine and C2C12 cells were transfected with Lipofectamine 2000 to induce transient expression of the indicated plasmids and siRNAs.

### siRNA Sequences

The small interfering RNAs (siRNA) for PLD1, PLD2 or controls (Luciferase) were purchased from Dharmacon Research, Inc. and used as previously described [41]. The results of a BLAST search of all siRNA sequences revealed no significant homology with any other sequences in the database program.

### Construction and Preparation of GST Fusion Proteins

GST fusion proteins of PLD1 were generated as previously described [41]. The individual fragments of PLD1 were then amplified by PCR using specific primers. They were digested with the restriction enzymes EcoRI and XhoI and ligated into pGEX-4T1 vector. Next, E. coli BL21 cells were transformed with individual expression vectors encoding the GST fusion proteins and expression was then induced by culture in the presence of 0.5 mM isopropyl-β-D-thiogalactopyranoside (IPTG) at 25°C for 4 h. After harvesting cells, GST fusion proteins of PLD1 were purified as previously described [41].

### Co-Immunoprecipitation

HEK-293 cells were cultured, harvested and lysed with PLD assay buffer (50 mM Hepes/NaOH, pH 7.4, 3 mM EGTA, 3 mM CaCl_2,_ 3 mM MgCl_2_ and 80 mM KCl) containing 1% Triton X-100, 1% cholic acid, 1 mM PMSF and protease inhibitor cocktail. After brief sonication, lysates were centrifuged at 100,000×g for 30 min and the supernatants (2 mg) were then incubated with anti-PLD-conjugated protein A Sepharose for 4 h. Co-immunoprecipitated complexes were washed three times with the same buffer and then analyzed by SDS-PAGE. The protein concentration was determined by Bradford assay.

### Immunoblot Analysis

Immunoblot analysis was performed as previously described [42]. Briefly, proteins were denatured by incubation at 95°C for 5 min in Laemmli sample buffer. The denatured proteins were then separated by SDS-PAGE and transferred to nitrocellulose membranes. After blocking in TTBS buffer (10 mM Tris/HCl, pH 7.5, 150 mM NaCl and 0.05% Tween 20) containing 5% skimmed milk powder, the membranes were incubated with individual monoclonal or polyclonal antibodies and subsequently reincubated with either anti-mouse or anti-rabbit IgG coupled with horseradish peroxidase. Detection was performed using an enhanced chemiluminescence (ECL) kit according to the manufacturer's instructions.

### Measurement of PLD Activity in Cells

PLD activity was assayed by measuring the formation of phosphatidylbutanol (PBtOH), which is the product of PLD-mediated transphosphatidylation, in the presence of 1-butanol [12]. Cells were labeled with [^3^H]myristic acid (10 µCi/ml) for 4 h and then washed twice with PBS. The labeled cells were maintained in glucose free-DMEM for 2 h at the indicated glucose concentrations and then incubated with 0.4% 1-butanol for 5 min. Next, cells were harvested into 0.8 ml of methanol and 1 M NaCl (1∶1) and then mixed with 0.4 ml of chloroform. After vortexing, the samples were centrifuged at 15,000×*g* for 1 min, the organic phase was dried and the lipids were separated using Silica Gel 60 TLC plate. Finally, the plates were developed with ethyl acetate/trimethylpentane/acetic acid (9/5/2  =  v/v/v). The amount of [^3^H]phosphatidyl-butanol was expressed as a percentage of the total [^3^H]-lipid to account for differences in cell labeling efficiency.

### *In Vitro* Phosphorylation Assay

AMPK was incubated with purified PLD1 (100 ng), GST-truncated PLD1 fusion mutants (1 µg) or precipitate complexes at 37°C for 15 min in kinase buffer (50 mM Tris/HCl, pH 7.4, 1 mM dithiothreitol, 0.02% Brij-35 and 10 µCi of [γ-^32^P]ATP (3,000 Ci/mmol)) in the presence of AMP (200 nM). An *in vitro* phosphorylation assay was then performed as previously described [29], [43]. The assay was terminated by the addition of 15 µl of 2×SDS sample buffer and boiling of the samples for 5 min. The sample was then subjected to SDS-PAGE and exposed to photographic film for autoradiography. The protein concentration was determined by Bradford assay.

### *In Vivo* Phosphorylation Assay

Initially, 1×10^7^ HEK-293 cells/100-mm dish were incubated with 3 mCi of [^32^P] orthophosphate in 5 ml of phosphate-free DMEM for 5 h at 37°C. Cells were then washed with PBS and treated with 50 mM 2-DOG for 10 min. Next, cells were washed with ice-cold isotonic buffer containing phosphatase inhibitor (30 mM NaF, 1 mM Na_3_VO_4_ and 30 mM Na_4_O_7_P_2_) and then lysed with 1 ml of lysis buffer (10 mM Tris/HCl, pH 7.5, 1 mM EDTA, 0.5 mM EGTA and 10 mM NaCl) containing 1% Triton X-100, 1% cholic acid, protease inhibitors (0.5 mM phenylmethylsulfonyl fluoride, 1 µg/ml leupeptin, 5 µg/ml aprotinin) and phosphatase inhibitors (30 mM NaF, 1 mM Na_3_VO_4_ and 30 mM Na_4_O_7_P_2_). After centrifugation (15,000×g for 15 min), equal amounts of soluble extract were incubated with 2 µg of anti-PLD antibody and 30 µl of immobilized protein A. The immunoprecipitated proteins were then separated by SDS-PAGE, transferred to nitrocellulose membranes and exposed to photographic film for autoradiography. The protein concentration was determined by Bradford assay.

### Determination of ^14^C-Glucose Uptake in Cells

C2C12 cells (2×10^5^) or L6 GLUT4-myc myoblast cells (2×10^5^) were seeded in a 6-well plate. After differentiation of these cells, no significant differences of cell numbers were observed in each well. Morphological changes were confirmed to determine the differentiation. There was no significant cell death or proliferation during ^14^C-glucose uptake assay. ^14^C-glucose uptake was determined as previously described [26]. Briefly, the differentiated C2C12 cells or L6 GLUT4-myc myotube cells were serum-starved for 2 h prior to the assay. Cells were then washed twice with Krebs-Henseleit buffer (NaCl 118, NaHCO_3_ 25, KCl 4.6, MgSO_4_ 1.2, KH_2_PO_4_ 1.2 and CaCl_2_ 2.5 mmol/l) and stimulated with or without the indicated agents. After incubation with 0.1 mCi/ml 2-deoxy[^14^C-glucose] at room temperature for 20 min, the uptake of ^14^C-glucose was terminated by washing cells three times with ice-cold PBS. Cells were then solubilized with 0.5 N NaOH and 0.1% SDS and their radioactivities were detected by scintillation counting. Each value was normalized to the total protein concentration. After the ^14^C-glucose uptake assay, the protein concentration was measured by Bradford assay and no significant differences were observed among samples. All assays were performed in duplicate.

### Measurement of the Cell Surface GLUT4 in Differentiated L6 GLUT4-myc Myotube Cells

L6 GLUT4-myc myoblast cells were seeded at 2×10^4^ per well in a 24-well plate. After differentiation of these cells, no significant differences in cell numbers were among wells. There was also no significant cell death or proliferation observed during the cell surface GLUT4 assay. Quantitative analysis of the GLUT4 on the cell surface was measured by an antibody coupled-colorimetric absorbance assay, as described previously [44]. Briefly, after stimulation, cells were exposed to anti-myc antibody (1∶100) for 1 h, fixed with 4% paraformaldehyde (PFA) for 10 min and then incubated with HRP-conjugated goat anti-rabbit IgG (1∶1,000) for 1 h. Next, cells were washed six times with PBS and then 1 ml of OPD reagent (0.4 mg/ml/o-phenylenediamine dihydrochloride and 0.4 mg/ml urea hydrogen peroxide) was added to each sample. The sample was incubated for 30 min at room temperature and the reaction was then stopped by the addition of 0.25 ml of 3 N HCl. The optical absorbance of the supernatant was measured at 492 nm. The background absorbance was determined using samples incubated in the presence of HRP-conjugated anti-rabbit IgG alone (without primary antibody) and then subtracted from the measured values. The protein concentration was measured by Bradford assay after cell surface GLUT4 assay and there were no significant differences observed among samples. All assays were performed in triplicate.

### Statistical Analysis

The results are expressed as the mean ± SE of data obtained from the indicated number of experiments performed. Statistical significance was determined using a Student's *t* test.
